# Effect of TALEN-mediated IL-6 knockout on cell proliferation, apoptosis, invasion and anti-cancer therapy in hepatocellular carcinoma (HCC-LM3) cells

**DOI:** 10.18632/oncotarget.20946

**Published:** 2017-09-15

**Authors:** Peng-Yuan Zhuang, Ke-Wei Zhang, Jian-Dong Wang, Xue-Ping Zhou, Ying-Bin Liu, Zhi-Wei Quan, Jun Shen

**Affiliations:** ^1^ Department of General Surgery, Xinhua Hospital, School of Medicine, Shanghai Jiao Tong University, Shanghai, People's Republic of China

**Keywords:** hepatocellular carcinoma, IL-6, TALEN

## Abstract

**Purpose:**

To determine the exact effect of Interleukin-6 (IL-6) on tumor cell proliferation, apoptosis, invasion, and anti-cancer therapy in hepatocellular carcinoma (HCC).

**Experimental Design:**

IL-6 was disrupted by transcription activator-like effector nucleases (TALEN) in HCCLM3 cells, and was used to evaluate the role of IL-6 on tumor cell proliferation, apoptosis, invasion and key signaling pathways involved in sorafenib and/or IFNα therapy.

**Results:**

IL-6 has no direct effect on cell proliferation and invasion but promotes cell apoptosis and up-regulate IL-33 and VEGF-A expression. IL-6 could attenuate the anti-proliferation effect by sorafenib and combination therapy but facilitate the pro-apoptosis of the combination therapy and augment the pro-invasive effect induced by single treatment. IL-6 could down-regulate p-STAT3, however up-regulate the p-MEK/p-ERK and NF-kB/iNOS expression, and it also facilitated the promotion on p-JAK2 and p-MEK/p-ERK by either sorafenib or IFN-α. *in vivo* study, IL-6 significantly promotes tumor growth. The combination treatment showed the highest inhibition on tumor growth which is derived from HCCLM3-IL6(-) cells.

**Conclusions:**

IL-6 has no direct effect on cell proliferation and invasion but promotes tumor cell apoptosis *in vitro* study. Sorafenib and combination therapies are suitable for HCC cells with low or no IL-6 expression confirmed *in vivo* study.

## INTRODUCTION

Our previous studies suggested that IL-6 was proven to be an important factor in tumor growth and metastasis [1]. The genetic signature derived from non-tumor liver tissue may reflect the promoting effects of IL-6 on the development of metachronous tumors that are independent from the primary resected HCC [2]. However, the exact effect and mechanism of the IL-6 was not well-elucidated in HCC. Therefore, our present study was conducted to study the exact effect of IL-6 on tumor cell proliferation, apoptosis, invasion, and related biological HCC cell behavior based on the knock-out of IL-6 by TALEN.

Although a variety of treatment modalities for HCC are currently available, drugs, such as sorafenib, and their multiple targets are one of the most relevant drugs for targeted therapies that could inhibit tumor growth and metastasis [3, 4]. However, although recognized targets, such as p-MEK/p-ERK signaling pathway, as well as VEGFR and PDGFR, which could be directly and indirectly modulated by sorafenib to inhibit tumor growth, distinct therapeutic value in individual patient could still be observed that may be caused by the different targets in different patient groups [5]. IL-6 plays a role in primary tumor progression, which induced MEK/ERK and JAK2/STAT3 signaling pathways lead to cell proliferation, apoptosis, angiogenesis; However, direct proof of the impact of IL-6 on therapy target of sorafenib treatment such as MEK/ERK and JAK2/STAT3 is missing and further studies in which IL-6 knockout is conducted and its impact on HCC cell behavior and anti-cancer effect needs to be tested.

Meanwhile, IFN-α has a variety of biological properties, including antiviral, immunomodulatory, antiproliferative, and antiangiogenic effects [6, 7]. Previous studies showed that IFN-α has inhibitory effect on HCC growth because of the antiangiogenesis by the down-regulation of VEGF-A [8–10]. Moreover, IFN-α was discovered to be related to its complicated mechanism, which could be exerted in different therapy values in patients with different IL-6 expressions [11]. however, the specific mechanism of IL-6 involved in anti-cancer therapy by IFN-α was not well elucidated. Therefore, IL-6, as an important factor in tumor growth and metastasis, should be further discovered its role in the mechanism of sorafenib or (and) IFN-α treatments of HCC.

TALEN is a gene editing tool with high efficiency and specificity and with low genotoxicity in targeted genome manipulation that cleaves the target genome and offers the advantage of achieving robust disruption of the target gene expression [12–14] In our present study, the HCCLM3, a human HCC cell line with high metastatic potential that originated from MHCC97, exhibits 100% transplant and metastatic abilities, as well as various manifestations reminiscent of tumor behavior in HCC patients, which were adopted in our previous studies [15]. Therefore, this study aims to know the role played by IL-6 in cell proliferation, invasion, apoptosis, and cytokine expression profiles, as well as its effect on sorafenib and IFNα therapies based on IL-6 knock-out by HCCLM3 cells.

## RESULTS

### Sorafenib and IFN-α had no direct effect on IL-6 expression in HCCLM3 cells

In the present *in vitro* study, we found that sorafenib and IFN-α had no obvious direct effect on IL-6 expression in HCCLM3 cells in both 24hr and 48hr, which was confirmed by RT-PCR (mean−△CT, −0.028±0.003 versus –0.032±0.004, *P* =.837 and −0.013±0.002 versus –0.015±0.001, *P* =.717 for 24hr and 48hr under sorafenib treatment respectively; −0.026±0.002 versus –0.028±0.002, *P* =.830 and −0.012±0.002 versus –0.013±0.001, *P* =.852 for 24hr and 48hr under IFN-α treatment respectively), Therefore, the research bias caused by the treatment itself on IL-6 expression could be removed and the exact effect of IL-6 on cell behavior and anti-cancer treatment could be determined.

### IL-6 knock-out had no effect on cell proliferation but enhanced the anti-proliferation effect by sorafenib and combination therapy

**Figure 1:**
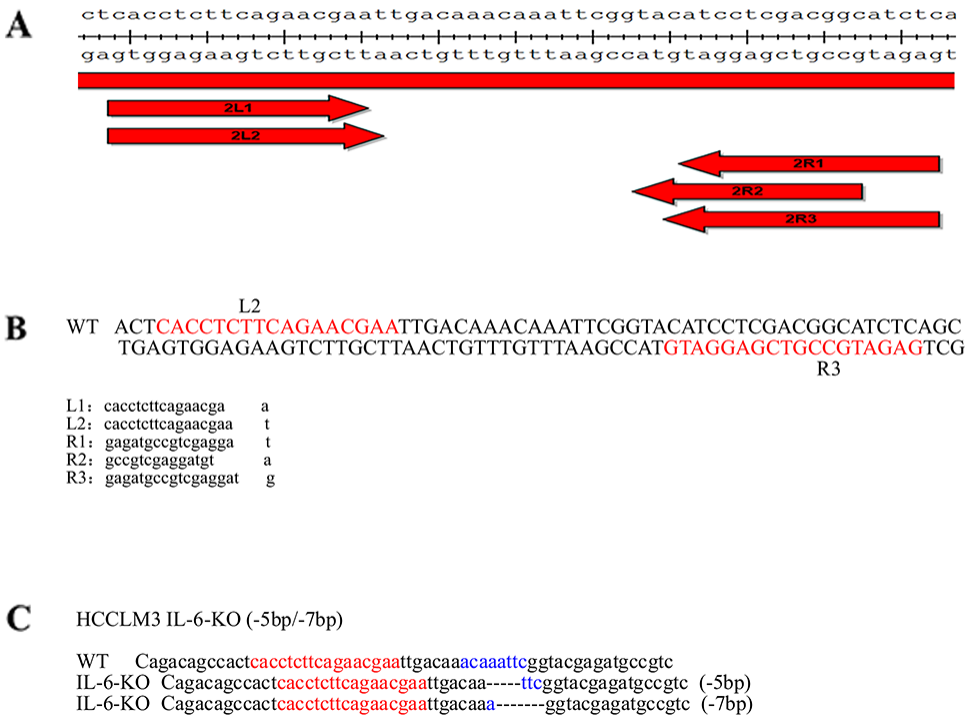
Stable cell line construction using TALENs **(A)** The TALEN design is in accordance to the sequence of IL-6. The arms of TALEN were designed as a 2×3 (2 left arms and 3 right arms) combination targets on the IL-6 (NCBI gene ID: 3569). The plasmids for the left and right arms of the TALENs were constructed using the FAST TALEN Kit (SIDANSAI, China). **(B)** After sequencing, five plasmids were transfected into HEK 293T cell lines using FuGene HD transfection reagent (Roche) in a 2×3 cross combination. A pair of TALEN (L2R3) plasmids was selected as the most effective knockout group after 3 days of puromycin screening and subsequent genomic PCR sequencing. **(C)** Mono-clone 25 exhibited bi-allelic IL-6 mutations. One allelic IL-6 was deleted at 5 bp, and the other was deleted at 7 bp on the same region.

**Figure 2:**
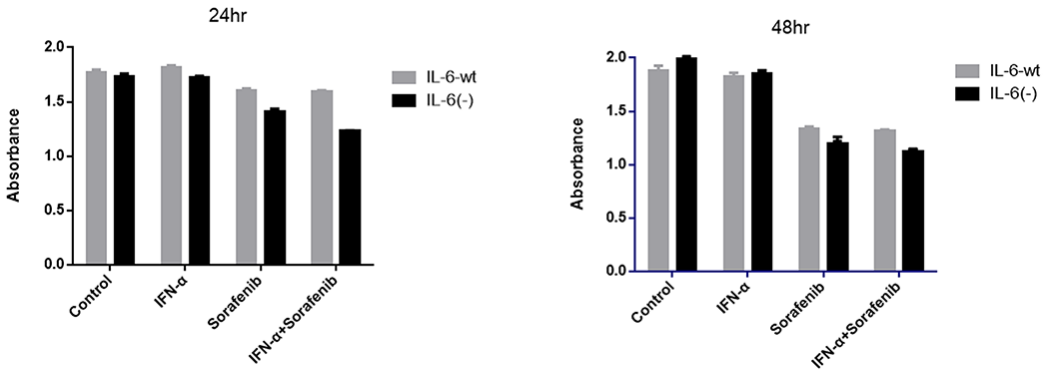
IL-6 knock-out had no effect on cell proliferation but enhanced the anti-proliferation effect by sorafenib and combination therapy No significant difference was observed in the proliferation between HCCLM3-wt and HCCLM3-IL6(-) cells for 24 and 48 hr. However, the proliferation of HCCLM3-wt cells could not be significantly inhibited by IFN-α and inversely inhibited by sorafenib. The inhibitory effect was not distinctly enhanced by the co-treatment of IFN-α and sorafenib. IL-6 attentuated the anti-proliferative effect of sorafenib as well as the co-treatment of sorafenib and IFN-α for 24 and 48 hr. Cell proliferation was evaluated by CCK-8 assay.

Based on IL-6 disruption by TALEN (Figure 1A–1C) in HCCLM3 cells, no significant difference was observed in the proliferation between HCCLM3-wt and HCCLM3-IL6(-) for 24 and 48 hr in the present study. However, the IL-6 knock-out has a distinct effect on the anti-proliferation therapy by IFN-α and sorafenib, that is, the proliferation of HCCLM3-wt cells could not be significantly inhibited by IFN-α and inversely inhibited by sorafenib. The inhibitory effect was not distinctly enhanced by the co-treatment of IFN-α and sorafenib. On the contrary, when IL-6 was knocked out, HCCLM3-IL6(-) still had no significant response to IFN-α but was more sensitive to the sorafenib treatment compared with HCCLM3-wt cells, especially the co-treatment of sorafenib and IFN-α for 24 and 48 hr, that is, 1.60 ± 0.02 versus 1.41 ± 0.02 (*P* =.012) and 1.33 ± 0.02 versus 1.19 ± 0.06 (*P* =.023) for HCCLM3-wt and HCCLM3-IL6(-) under the sorafenib treatment for 24 and 48 hr, respectively,; and 1.59 ± 0.02 versus 1.22 ± 0.01 (*P* =.035) and 1.31 ± 0.01 versus 1.11 ± 0.03 (*P* =.027) for HCCLM3-wt and HCCLM3-IL6(-) under co-treatment for 24 and 48 hr, respectively. Cell proliferation was evaluated by CCK-8 assay (Figure 2).

### IL-6 knock-out attenuated side pro-invasive effect induced by the single treatment of either sorafenib or IFN-α

In the present study, no significant difference was found in the cell invasion capacity between HCCLM3-wt and HCCLM3-IL6(-) for 24 and 48 hr. However, under sorafenib or IFN-α treatment, the cell invasion capacity was significantly changed in 24 and 48 hr.

**Figure 3:**
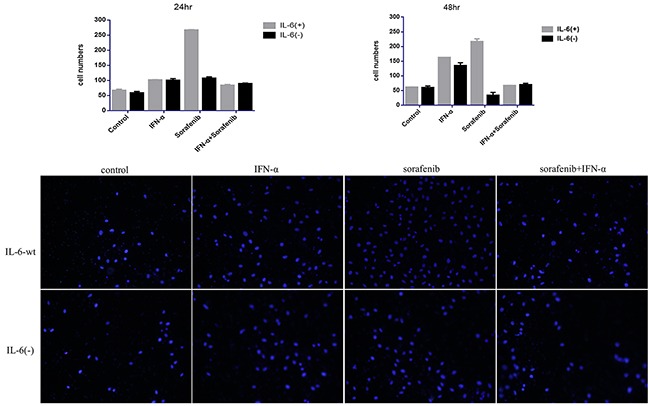
IL-6 knock-out attenuated side pro-invasive effect induced by the single treatment of either sorafenib or IFN-α IL-6 had no effect on tumor invasion; the cell invasion capacity was significantly increased in 24 and 48 hr by single treatment of either sorafenib or IFN-α treatment, which was not observed in the co-treatment group; IL-6 knock-out could attenuate the pro-invasive side effect of single treatment; however the effect was not observed when co-treatment was conducted.

Our previous study shows that sorafenib could promote HCCLM3-wt cell invasion and migration *in vitro* and *in vivo* [16], which was also confirmed by our present study (Figure 3), in which sorafenib prominently promoted the invasion in HCCLM3-wt cells in 24 and 48 hr (the cell numbers in control group versus sorafenib-treated group was 66.09 ± 4.72 versus 265.49 ±2.65 (*P* =.0170) and 59.92 ±2.09 versus 215.13 ±10.94 (*P* =.0169) for 24 and 48 hr, respectively). For HCCLM3-IL6(-) cells, the attenuated promotion effect was found in 24 hr and even decreased cell invasion capacity in 48 hr under sorafenib treatment (the cell numbers in the controls versus sorafenib-treated group was 57.85 ±5.88 versus 105.49 ±6.26 (*P* =.025) and 59.31 ±6.41 versus 33.19 ±10.04 for 24 and 48 hr (*P* =.034), respectively. The results indicated that the promoted invasion capacity exerted by sorafenib was more prominent in HCCLM3-wt cells than in HCCLM3-IL6(-) cells. Moreover, IL-6 knock-out may attenuate the pro-invasion side effect of sorafenib.

In terms of IFN-α, although the IFN-α treatment in our previous study did have insignificant effect on HCC-LM3 cell migration and invasion, a trend in the pro-invasive effect on HCC-LM3 cells can still be observed. Thus, in our present study, the adaptive dose of IFN-α of 50000 U/mL with minimal cell cytotoxicity in cell transwell assay was adopted and found the significant pro-invasive effect in the HCCLM3-wt cells in 24 and 48 hr, and the cell numbers in the controls versus IFN-α-treated group was 66.09 ±4.72 versus 100.42 ±2.32 (*P* =.017) and 59.92 ±2.09 versus 161.23 ±1.81 for 24 and 48 hr (*P* =.034), respectively. The effect was also attenuated by IL-6 knock-out in 48 hr, that is, the cell numbers in the controls versus IFN-α-treated group were 59.32 ±6.40 versus 133.79 ±10.7 (*P* =.017) for 48 hr (*P* =.034), respectively.

Furthermore, although a slightly pro-invasive effect was observed (the cell numbers in the controls versus co-treated group was 66.09 ±4.72 versus 82.15±4.25(*P* =.083), and 59.92 ±2.09 versus 65.97 ±1.74 (*P* =.065), for 24 and 48 hr, respectively), the pro-invasive side effect prominently decreased compared with the single treatment group once the combination of IFN-α and sorafenib was adopted in HCCLM3-wt cells. However, IL-6 knock-out has no significant effect on the cell invasion capacity compared with HCCLM3-wt (the cell numbers in the controls versus co-treated group were 57.85 ±5.88 versus 87.83 ±3.53, *P* =.089 and 59.31 ±6.40 versus 68.89 ±5.84, *P* =.065, for 24 and 48 hr, respectively), which indicated that the co-treatment has no significant side promotion effect on the cell invasion capacity, compared with the single treatment of either IFN-α or sorafenib. Furthermore, although IL-6 knock-out could attenuate the pro-invasive effect induced by the single treatment, the effect was not observed when co-treatment was conducted.

### IL-6 promotes tumor cells apoptosis and enhance the pro-apoptosis effect by combining sorafenib and IFN-α

**Figure 4:**
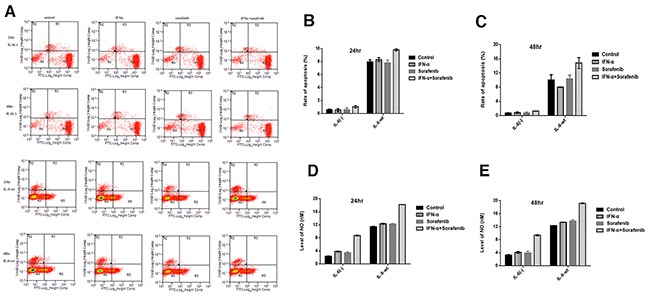
IL-6 promotes tumor cells apoptosis and enhance the pro-apoptosis effect by combining sorafenib and IFN-α **(A-C)** The cell apoptosis rate of HCCLM3-wt cells was significantly higher than that of HCCLM3-IL6(-) cells for 24 and 48 hr. No significant effect on cell apoptosis was found by either sorafenib or IFN-α in both cell lines. However, the prominently increased cell apoptosis by co-treatment was observed and was significantly attenuated in HCCLM3-IL6(-) cells. **(D-E)** Higher NO levels was observed in HCCLM3-wt cells as compared with HCCLM3-IL6(-) cells, and highest was observed in combination treatment.

Cell apoptosis was measured over a period of 24 and 48 hr under similar conditions (Figure 4A–4C). The cell apoptosis rate of HCCLM3-wt cells was significantly higher than that of HCCLM3-IL6(-) cells when cultured in the serum-supplemented medium for 24 and 48 hr (the cell apoptosis rate in the HCCLM3-wt cells versus HCCLM3-IL6(-) cells group was 7.99 ± 0.30 versus 0.56 ± 1.13 for 24 hr (*P* =.002) and 10.06 ±1.44 versus 0.66 ± 0.10 for 48 hr (*P* =.0015)). No significant effect on cell apoptosis was found by either sorafenib or IFN-α in both cell lines. However, the prominently increased cell apoptosis by co-treatment was observed and was significantly attenuated in IL6(-) HCCLM3 cells, that is, the cell apoptosis rates in the HCCLM3-wt cells versus HCCLM3-IL6(-) cells was 9.795 ± 0.18 *v*ersus 1.02 ± 0.19 for 24 hr (*P* =.0013) and 14.8 ±1.55 versus 1.21 ± 0.02 for 48 hr (*P* =.0011). The cell apoptosis assay indicated that IL-6 expression could promote cell apoptosis and may be an excellent target for the co-treatment of sorafenib and IFN-α.

### IL-6 knock-out down-regulate the tumor derived IL-33 and VEGF-A expression

**Figure 5:**
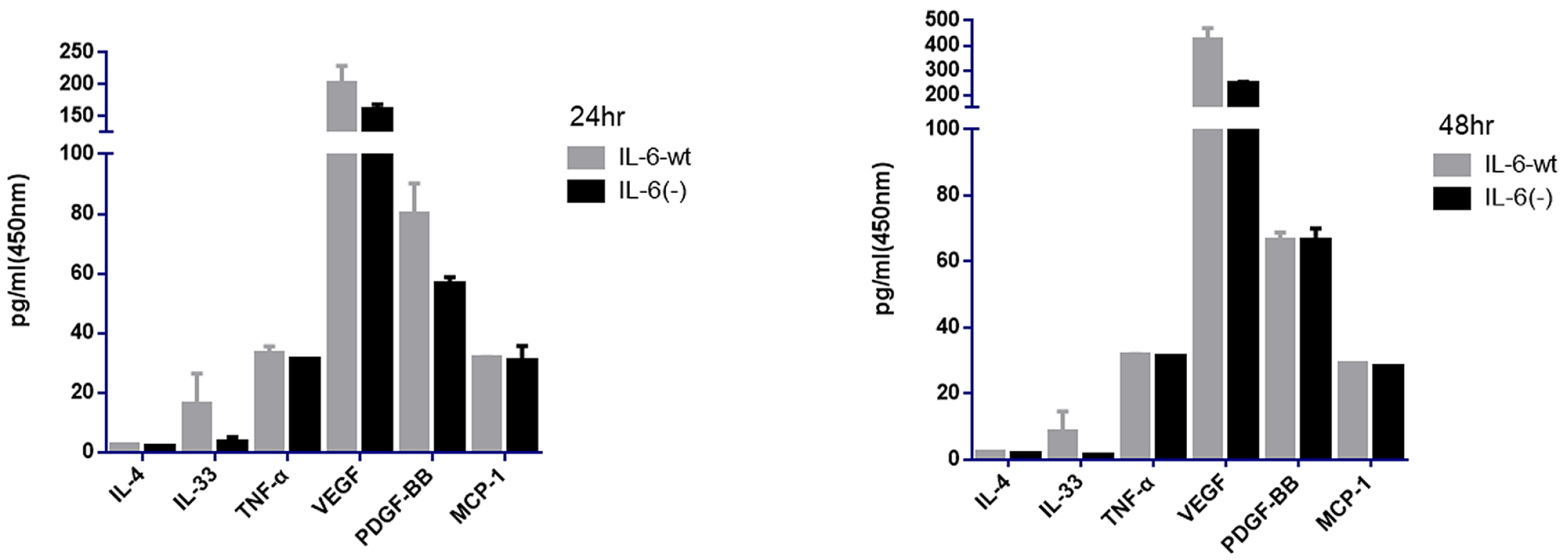
IL-6 knock-out down-regulate the tumor derived IL-33 and VEGF-A expression The supernatant of tumor cell culture was assessed by using ELISA assay. We found that the IL-4, TNF-α, MCP-1, PDGF-BB levels was similar in both 2 cell lines. However, IL-33 and VEGF-A were significantly decreased in the HCCLM3-IL6(-) cells for 24 hr and 48 h, respectively.

Macrophage has a great role in HCC recurrence and metastasis. Moreover, the M1 and M2 phenotypes of macrophage, as well as its distinct function, could be transformed interchangeably [17, 18]. Previous studies found that IL-33 and IL-4 could promote macrophage transformation from M1 phenotype to M2 phenotype, and TNF-α could transform M2 into M1 phenotype [19]. Tumor cell-derived chemokines, such as MCP-1, could contribute to the macrophage infiltration into tumor tissue and exerted its effect on tumor growth and metastasis [20, 21]. Furthermore, important angiogenesis factors, such as VEGF-A and PDGF-BB secreted by tumor cells, have important roles in tumor angiogenesis and recurrence [22, 23]. The supernatant of the tumor cell culture was assessed using ELISA assay to determine whether the IL-6 knock-out has an effect on macrophage polarization and migration as well as in tumor angiogenesis (Figure 5). The IL-4, TNF-α, MCP-1, and PDGF-BB levels were similar in both cell lines. However, IL-33 level significantly decreased in the HCCLM3-IL6(-) cells compared to the HCCLM3-wt cells (3.06± 2.13 versus 15.8± 10.6 (*P* =.015) and 1.21±0.08 versus 8.23± 6.50 (*P* =.023) for 24 and 48 hr, respectively). The angiogenesis factor VEGF-A significantly decreased in the HCCLM3-IL6(-) cells compared with HCCLM3-wt cells (158.45 ± 10.75 versus 199.57± 29.62 (*P* =.031) and 241.64 ± 12.88 versus 416.99± 53.05 (*P*=.026) for 24 and 48 hr, respectively. The ELISA assay indicated that IL-6 knock-out could down-regulate IL-33 and VEGF-A secretion, which may restore M1 phenotype and inhibit tumor angiogenesis, growth, and recurrence.

### IL-6 on signaling transduction pathways including p-MEK/p-ERK, p-JAK2/p-STAT3, and NF-kB-iNOS

**Figure 6:**
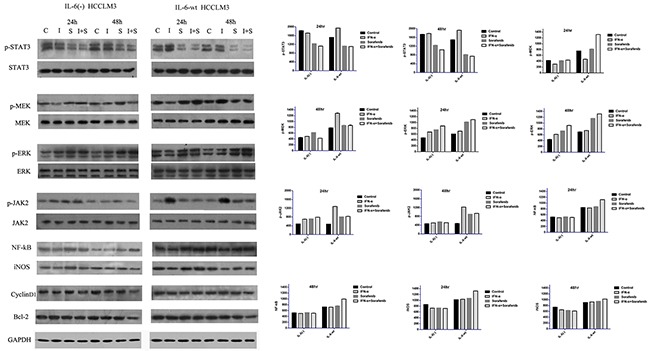
IL-6 on signaling transduction pathways including p-MEK/p-ERK, p-JAK2/p-STAT3, and NF-kB-iNOS Three major signaling transduction pathways, including p-MEK/p-ERK, p-JAK2/p-STAT3, and NF-kB-iNOS were tested to explore the downstream modulation of IL-6. IL-6 could down-regulate the protein expression of p-STAT3 in a time-dependent manner, However, IL-6 has no effect on p-JAK2 expression. IL-6 has a promotion effect on the expression of p-MEK/p-ERK and NF-kB/iNOS. Furthermore, other proliferation- and apoptosis-related protein, such as cyclin-D1 and Bcl-2, were not affected by the IL-6. We found that the p-STAT3 expression was similarly reduced by the sorafenib in both cell lines in 24 hr and significantly reduced in 48 hr in HCCLM3-wt cells. p-JAK2, p-MEK and p-ERK were inversely up-regulated by sorafenib in a time-dependent manner. IFN-α had the opposite promotion effect on p-JAK2/p-STAT3 and p-MEK/p-ERK. The effect of co-treatment on these three major signaling transduction pathways is similar as the sorafenib group in terms of p-JAK2/p-STAT3 and p-MEK/p-ERK. In terms of NF-kB/iNOS, either IFN-α or sorafenib has no significant effect on the cell signal pathways. However, both statistically increased in the combination group in HCCLM3-wt cells in 48 hr.

Three major signaling transduction pathways, including p-MEK/p-ERK, p-JAK2/p-STAT3, and NF-kB-iNOS (Figure 6), were tested to explore the downstream modulation of IL-6 and found that IL-6 could down-regulate the protein expression of p-STAT3 in a time-dependent manner. However, IL-6 has no effect on p-JAK2 expression. On the contrary, IL-6 has a promotion effect on the expression of p-MEK/p-ERK and NF-kB/iNOS. Furthermore, other metastasis-related proteins, such as MMP-9, MMP-2, and proliferation- and apoptosis-related protein, such as cyclin-D1 and Bcl-2, were not affected by the IL-6 knock-out.

Next, the effect of sorafenib treatment on the three major signaling transduction pathways of the two cell lines was tested. We found that the p-STAT3 expression was similarly reduced by the sorafenib in both cell lines in 24 hr and significantly reduced in 48 hr in HCCLM3-wt cells. Sorafenib could increase p-JAK2 expression in a time-dependent manner, that is, the p-JAK2 expression in 48 hr was higher in IL-6-wt cells. Furthermore, p-MEK and p-ERK were inversely up-regulated by sorafenib in a time-dependent manner and was higher in 48 hr in HCCLM3-wt cells.

Furthermore, the effect of IFN-α treatment on the two signaling transduction pathways of both cell lines were tested. We found that IFN-α had the opposite promotion effect on p-JAK2/p-STAT3 and p-MEK/p-ERK; moreover, the latter showed a time-dependent manner in HCCLM3-wt cells. However, the up-regulation effect was considerably attenuated when IL-6 was knocked out, that is, the similar or slightly higher expression of these factors was observed and compared with the control group.

The effect of IFN-α and sorafenib co-treatment on these three major signaling transduction pathways is similar to the expression of the sorafenib group in terms of p-JAK2/p-STAT3 and p-MEK/p-ERK. In terms of NF-kB- iNOS, either IFN-α or sorafenib has no significant effect on the cell signal pathways. However, both statistically increased in the combination group in HCCLM3-wt cells.

The amount of NO using a NO-sensitive electrode showed that HCCLM3-wt cells produced higher NO levels as compared with HCCLM3-IL6(-) cells. Moreover, as compared with control group, the combination treatment increased NO production, and either treatment of IFN-α or sorafenib group has no difference among the three groups (Figure 4D–4E).

### IL-6 on tumor growth and treatment *in vivo*

**Figure 7:**
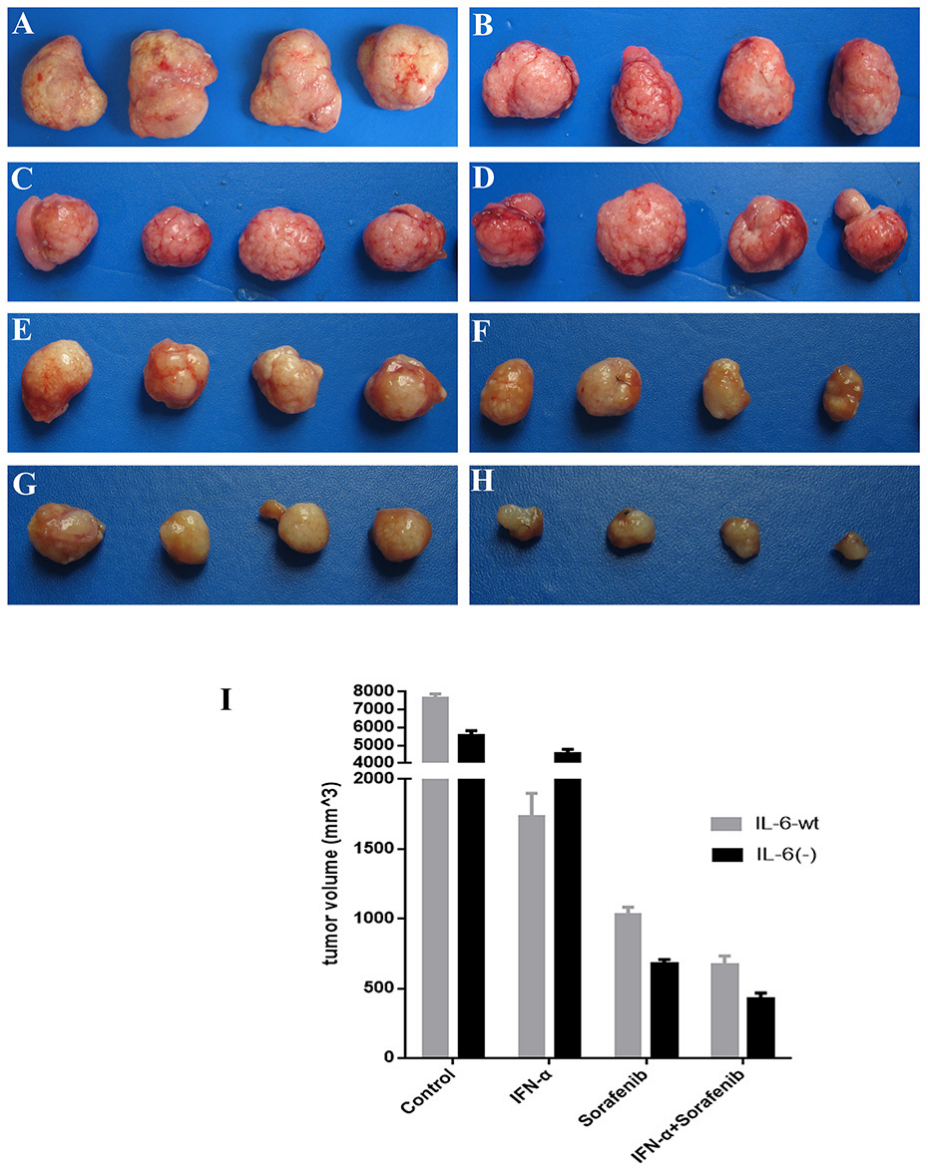
IL-6 on tumor growth and treatment *in vivo* IL-6 significantly promote tumor growth *in vivo* study for six wk as compared with HCC with HCCLM3-IL6(-) cells. Sorafenib has predominant inhibition on tumor growth when IL-6 was knocked out, whereas IFN-α treatment significantly inhibited tumor growth in the HCC derived from HCCLM3-wt cells. The combination of sorafenib and IFN-α treatment showed the highest predominant inhibition on tumor derived from HCCLM3-IL6(-) cells. **(A)** Control group (HCCLM3-wt); **(B)** control group (HCCLM3-IL-6(-)); **(C)** IFN-α treatment group (HCCLM3-wt); **(D)** IFN-α treatment group (HCCLM3-IL-6(-)); **(E)** sorafenib treatment group (HCCLM3-wt); **(F)** sorafenib treatment group (HCCLM3-IL-6(-)); **(G)** combination treatment group (HCCLM3-wt); **(H)** combination treatment group (HCCLM3-IL-6(-)).

Our results *in vivo* (Figure 7) showed that IL-6 significantly promote tumor growth *in vivo* compared with HCC with HCCLM3-IL6(-) cells (7659.0±215.4 mm^3^ versus 5567.4±254.4 mm^3^, *P*=.013). Sorafenib has predominant inhibition on tumor growth when IL-6 was knocked out (1026.6±56.0 mm^3^ versus 673.4±34.0 mm^3^, *P*=.027) based on treatment, whereas IFN-α treatment significantly inhibited tumor growth in the HCC derived from HCCLM3-wt cells (4565.2±213.3 mm^3^ versus 1730.2±169.3 mm^3^, *P* =.021). The combination of sorafenib and IFN-α treatment showed the highest predominant inhibition on tumor derived from HCCLM3-IL6(-) cells (422.2±45.0 mm^3^ versus 668.6±65.0 mm^3^, *P* =.034). All treatments did not induce any significant body weight loss.

## DISCUSSION

IL-6 plays a role in primary tumor progression associated with MEK/ERK and JAK2/STAT3 signaling pathways and its exact role in cell behavior of hepatocellular carcinoma and effect on the anti-cancer therapy such as sorafenib and IFN-α treatments was still not well elucidated; The present study shows that IL-6 has different effects on cell proliferation, apoptosis, and invasion in HCCLM3, as well as in cytokines and related cell signaling pathway secretions and sorafenib and IFN-α treatments.

IL-6 has no significant effect on tumor cell proliferation compared with other tumors derived from IL-6 drive cell proliferation [24, 25]. Further research found that IL-6 has no significant response to IFN-α treatment, which was in accordance with our previous study, which states that IFN-α has no direct effect on HCCLM3 cells *in vitro* [26]. However, sorafenib has significant inhibitory effect on both cell lines. Great impact was observed when IL-6 was knocked-out. Furthermore, the combination therapy of IFN-α and sorafenib could augment the inhibition effect on the proliferation of both cell lines In conclusion, sorafenib and combination therapies are suitable for HCC cells with low or no IL-6 expression.

On the aspect of cell proliferation, sorafenib significantly inhibited the proliferation of both cell lines in 24 and 48 hr and has significant effect on its therapy targets in both cells lines. The present study found that the mechanism involved in the cell signal pathway associated with sorafenib treatment may be caused by the balance of the effects on both major cell signaling pathways: one was the up-regulation of p-MEK and p-ERK, and the other was the down-regulation of p-JAK/p-STAT3. The balance between these two pathways may decide its effect on cell proliferation.

IL-6 presents its negative effect on the p-STAT3 in a time-dependent manner, and IL-6 knock-out resulted in significantly high p-STAT3 expression in 48 hr. However, contrary to our study [24, 25], the other studies showed that the IL-6 could result in tyrosine phosphorylation and increased STAT3 activity.

Sorafenib has insignificant effects on IL-6 expression in HCCLM3 cell, which helps observe the exact effect of sorafenib on HCCLM3 cell proliferation. In terms of p-STAT3, IL-6 in HCCLM3 cell was similarly reduced by sorafenib in both cell lines in 24 hr and significantly reduced in 48 hr in HCCLM3-wt cells compared with HCCLM3-IL6(-) cells. IL-6 knocked-out effectively attenuated the sorafenib-induced down-regulation of p-STAT3 expression in 48 hr, which may be the effect of sorafenib on p-STAT3 depending on the basic expression of p-STAT3 associated IL-6, that is, the time-dependent up-regulation of p-STAT3 by IL-6 knocking-out. The affirmative inhibition effect of sorafenib on p-STAT3 was observed in our study. The present study found that IL-6 expression did not affect p-JAK2. However, sorafenib could increase p-JAK2 expression in a time-dependent manner. The similar p-JAK2 expressions between the cell lines in 24 hr and a high p-JAK2 expression in IL-6-wt cells under sorafenib suggest that IL-6 could enhance the up-regulation of p-JAK2 expression driven by sorafenib.

The unsynchronized p-JAK2/p-STAT3 cell signal transduction, in response to the sorafenib, may be associated with the negative feedback regulation between these two factors. However, the biological function caused by the downstream signaling pathways may depend mainly on p-STAT3 expression. Therefore, in our study, the p-STAT3 expression was considered for subsequent biological behavior, such as tumor cell proliferation.

IL-6 was previously shown to activate the MEK/ERK-signaling pathway in various kinds of cells [27–29] and we also found that IL-6 could up-regulate p-MEK and p-ERK expressions without treatment intervention in 24 and 48 hr in a time-dependent manner. However, compared with other studies, p-MEK and p-ERK were inversely up-regulated by sorafenib in a time-dependent manner and is predominant in HCCLM3-wt cells, which may be associated with high basic p-MEK and p-ERK expressions driven by IL-6 cells. However, the up-regulation of p-MEK and p-ERK by sorafenib in HCCLM3 cells was different from previous studies because sorafenib could directly suppress the proliferation of HCC cells by inhibiting RAF kinase and blocking the RAF/MEK/ERK signal pathway [30]. An opposite effect was observed in our present study; this effect may be compromised by complementary and/or feedback mechanisms, which may partially restore the levels of p-ERK [31, 32], that is, the inhibition of phospho-c-RAF Ser 259 by sorafenib facilitated the phosphorylation of c-Raf at Ser338 is possible, which in turn inversely promoted p-MEK and p-ERK [33]. However, previous studies showed that cross-phosphorylation between tyrosine and serine/threonine protein kinases may support the widespread crosstalk between ERK and JAK/STAT3 signaling pathways [34] once the inhibition of p-STAT3 by sorafenib was conducted. Thus, the negative feedback effect may be associated with the up-regulation of p-MEK and p-ERK. Other research also found that the blockage of MEK/ERK or PI3K/AKT separately can result in the activation of other pathways [35]. Moreover, the delicate mechanism was confirmed by our further studies.

Therefore, in 24 hr, a similar down-regulated p-STAT3 and a lower p-MEK and p-ERK in HCCLM3-IL6(-) cells by sorafenib result in greater inhibitory effect in cell proliferation than HCCLM3-wt cells; whereas in 48 hr, the relatively higher p-STAT3 expression in HCCLM3-IL6(-) cells may not counteract the negative effect caused by the relatively low expressions of other factors, such as p-MEK and p-ERK, and great inhibitory effect could also be observed in HCCLM3-IL6(-) cells in 48 hr.

Furthermore, although IFN-α had an effect on the two signaling transduction pathways of cell lines, its effect on the tumor cell proliferation was insignificant and was in accordance with our previous studies that showed that IFN-α has no significant effect on tumor cell proliferation *in vitro*. Moreover, the anti-tumor effect exerted *in vivo* studies may be associated with its indirect anti-angiogensis effect. Consequently, the present study showed that the combined therapy has a similar effect on the aforementioned signaling pathway compared with the sorafenib alone. Furthermore, no significant synergy effect was observed.

In terms of tumor cell invasion, IFN-α or sorafenib increased the invasion ability of tumor cells although IL-6 has insignificant effect on tumor cells. However, once IL-6 was knocked out, the tumor cell migration was greatly reduced even lower than the control group, which suggested that the increased cell migration may be associated with the cell signaling pathways by these therapies, and the effect of sorafenib was significantly greater than IFN-α, which may be associated with the up-regulation of p-MEK and p-ERK for sorafenib treatment and the up-regulation of p-JAK2/p-STAT3 for IFN-α, respectively. Considering that the target of each therapy may not be exactly the same when these two therapies were combined, the antagonistic effect, such as the down-regulation of p-STAT3 by sorafenib, and not the synergic ones were observed to counteract the up-regulation of p-STAT3 by IFN-α, which may result in similar cell invasion ability compared with control groups.

However, although IL-6 has no significant distinct effect on the tumor cell invasion, once the IL-6 was knocked out, the negative pro-invasive effect of the single treatment could be eliminated, which suggests that sorafenib is suitable for tumor cells with low IL-6 expression because of its ability to counteract the pro-invasive side effects by the anti-tumor therapy by the down-regulation of p-MEK and p-ERK when IL-6 was knocked-out. Furthermore, in terms of the treatment value of the combined treatment, the combination treatment of IFN-α and sorafenib is more applicable for tumor cells with normal IL-6 expression compared with the single treatment that could counteract the increased cell invasion ability related to either IFN-α or sorafenib treatment. In terms of MMP-9 and MMP-2, both were not up-regulated by sorafenib and IFN-α, and other factors associated with tumor invasion and migration require further confirmation in our future research.

In terms of tumor apoptosis, IL-6 promoted the apoptosis of HCCLM3 cells without any treatment and was presented in a time-dependent manner. The role of IL-6 in cell apoptosis was controversial in different studies, demonstrating that bipotential murine oval liver (BMOL) cell-secreted IL-6 could induce the apoptosis of activated hepatic stellate cell (HSC) by mediating the activation of NF-kB-iNOS-NO-ROS signaling [36]. Other studies found that the elemene could induce the apoptosis of gastric cancer cells associated with the increased expression levels of p-ERK protein and Bax mRNA, however the reduced level of Bcl-2 mRNA expression [37], in our research, we found IL-6 exerted the promotion effect on NF-kB/iNOS expression, high levels of NO production, as well as p-MEK/p-ERK in a time-dependent manner, which suggested that IL-6 promoted the apoptosis of HCCLM3 cells may through the activation of NF-kB/iNOS-NO and p-MEK/p-ERK.

However, other studies also found that opposite results in IL-6 could inhibit cell apoptosis by up-regulating p-STAT3Tyr705 expression and its direct target genes, the anti-apoptotic proteins Bcl-2, XIAP, and surviving, as well as by down-regulating the apoptosis proteins Bak [38]. Although the down-regulation of p-STAT3 in 48 hr by IL-6 may be associated with the partial role in IL-6 in the pro-apoptosis effect, the prominent cell apoptosis affected by IL-6 in 24 hr was already observed. Meanwhile, the p-STAT3 affected by IL-6 at this time was insignificant; therefore, the IL-6 mechanism on cell apoptosis might be complicated in multiple mechanisms, such as the activation of NF-kB/iNOS-NO and p-MEK/p-ERK and the down-regulation of p-STAT3 in 48 hr.

In terms of treatment on tumor apoptosis, although IFN-α or sorafenib could promote apoptosis compared with the control group, no significant difference was observed. Moreover, the synergistic effect on cell apoptosis in combination treatment was prominent in the HCCLM3-wt cells, which may be associated with the synergistic effect of the combination treatment on the aforementioned apoptosis-related cell signaling pathways. This finding suggests that the treatment effect on cell apoptosis was mainly on the basis of the role of IL-6. Therefore, HCCLM3 cells with normal IL-6 expression are suitable for combination therapy to promote cell apoptosis.

Furthermore, tumor cells that were secreted by cytokine expression profiles were changed when IL-6 was knocked out, that is, IL-33, which was expressed by tumor cells, was significantly reduced, suggesting that tumor cells secrete factors promoting M1 differentiation to M2 were greatly reduced, and M1 cells were subsequently reserved, which played the important role in inhibiting tumor growth. Meanwhile, the major angiogenic factor, VEGF-A, was also greatly reduced, which could result in an anti-angiogenesis effect. Therefore, the knock-out of IL-6 may be associated with inhibited tumor angiogenesis and tumor growth.

In conclusion, based on the *in vitro* study, IL-6 hasno significant effect on cell proliferation and invasion, but could exert pro-apoptosis effect and up-regulate tumor derived IL-33 and VEGF-A. In terms of IL-6 on treatment, sorafenib and combination therapies are suitable for HCC cells with low or no IL-6 expression because of its augmented anti-proliferation effect and attenuated side pro-invasion by the single treatment; however, the low cell apoptosis of the combination therapy in cell with low or no IL-6 expression may counteract the application of combination treatment. Therefore, the preliminary *in vivo* study was conducted to the evaluate the effect of IL-6 on tumor growth and treatment application. Although the pro-apoptosis effect exerted by IL-6 was found in *in vitro* study, IL-6 could promote tumor growth, which may be associated with the tumor environment, such as the aforementioned upregulated of IL-33, VEGF-A *in vitro* study, and other factors. In terms of treatment application, sorafenib is suitable for HCC with the low expression of IL-6, whereas IFN-α is suitable for HCC with high IL-6 expression, which may be caused by the up-regulation of NF-kB and thereby could facilitate the IFN-α treatment as found in other studies [11]. The combination treatment is applicable to HCC with the low or no expression of IL-6, and the specific mechanism requires further study.

## MATERIALS AND METHODS

### Stable cell line construction using TALENs

The TALEN design is in accordance to the sequence of IL-6. The arms of TALEN were designed as a 2×3 (2 left arms and 3 right arms) combination targets on the IL-6 (NCBI gene ID: 3569). The plasmids for the left and right arms of the TALENs were constructed using the FAST TALEN Kit (SIDANSAI, China). After sequencing, five plasmids were transfected into HEK 293T cell lines (Human Embryonic Kidney 293T cell lines) using FuGene HD transfection reagent (Roche) in a 2×3 cross combination. A pair of TALEN plasmids was selected as the most effective knockout group after 3 days of puromycin screening and subsequent genomic PCR sequencing. The HCCLM3 cell line was routinely cultured, as described, and was plated for 16 h before transfection. The HCCLM3 cell line was transfected with the indicated plasmids using Fugene HD (Roche) according to the instructions of the manufacturer. The selected pair of TALEN plasmids, which has the highest cleavage efficiency, was co-loaded into the HCCLM3 cell line. The amounts of plasmids per well for the 6-well plates included in each transfection were 2 ug of pTALEN-Left, 2 ug of pTALEN-Right, and 0.5 ug of pEGFP as a transfection marker. The cells were exposed to 2 μg/mL puromycin for 3 days. Then, the medium that contains puromycin was replaced with growth media. After a week of monoclonal culturing, the genomic DNA from IL-6-target and control cells was extracted and was then used to amplify the DNA fragment that contains the IL-6-targeted site. The amplifying fragment was identified using TA clone sequencing. Mono-clone 25 exhibited bi-allelic IL-6 mutations. One allelic IL-6 was deleted at 5 bp, and the other was deleted at 7 bp on the same region (Figure 1A–1C).

### Cells and drugs

HCCLM3-wt or HCCLM3-IL6(-) were grown as a monolayer culture in DMEM supplemented with 10% BSA. All the cells were cultured at 37 °C in a 5% CO_2_, 95% air environment in humidified incubators. Sorafenib was finally prepared to a 1× solution with cremophor EL/ethanol/water (12.5:12.5:75, vehicle solution) [39], and the concentration used in our present vitro studies was 10 umol/L, because the concentration of sorafenib in human plasma was between 5 and 7 mg/L, which is 7.8–10.9 umol/L in humans [40], and which was convertible to the dosage of 30 mg/kg *in vivo* study. Recombinant IFN-α (Sinogen, Kexing Bioproduct Company Ltd. Shenzhen, PR China) is a highly purified protein with a molecular weight of 19,400 Da. The protein is expressed by the gene isolated from leukocytes of healthy Chinese individuals [26].

### Cell proliferation assay

HCCLM3-wt or HCCLM3-IL6(-) was plated in triplicate into 96-well plates in DMEM. Cells were cultured for 24 and 48 hr to detect cells that respond to sorafenib (10umol/L) and IFNα (50000U/mL) treatments. The cell counting kit-8 (CCK-8) (Dojindo Laboratories, Kumamoto, Japan) was used to determine cell viability. A total of 20 μg of CCK-8 in phosphate buffered saline (PBS) was added to the plates. The absorbance of each well was read at 490 nm using a microplate reader (Nexcelom, Lawrence, MA). Culture medium (DMEM medium) without cells were used as blank control. All experiments were performed in triplicate.

### Apoptosis assay

HCCLM3-wt or HCCLM3-IL6(-) cells, which were cultured in serum-free DMEM with orafenib (10umol/L) and (or) IFNα (50000U/mL) or vehicle for 24 and 48 hr were collected and analyzed for the presence of apoptotic cells using Annexin V-FITC Apoptosis Detection Kit (BD Pharmingen, San Jose, CA) following the instruction of the manufacturer. Flow cytometry analysis was performed using FACS caliber cytometer (R&D Systems, Minneapolis, MN). Replicated assays were performed.

### Matrigel invasion assay

Tumor cell invasion was measured with a Biocoat Matrigel invasion chamber (Becton Dickinson Labware, Bedford, MA) according to the instructions of the manufacturer. Cells (6 × 10^4^) in 200 μL serum-free medium were seeded onto the upper chamber, and 600 μL DMEM containing 10% BSA was added to each well in the lower chamber. After incubation with Sorafenib (10 umol/L) and IFNα (50000 U/mL) and combination treatment, as well as vehicle, for 24 and 48 hr at 37 °C, the cells attached to the lower chamber were fixed with methanol, stained with Giemsa, and then were counted under a light microscope.

### ELISA

The ELISA of plasma protein levels of human origin, including IL-4, IL-33, TNF-α, MCP-1, PDGF-BB, and VEGF-A, was analyzed by ELISA using Quantikine ELISA kits (R&D Systems). All analyses were performed in duplicate.

### Western blotting

After 24 and 48 hr of culturing HCCLM3-wt or HCCLM3-IL6(-) with Sorafenib (10 umol/L) and IFNα (50000 U/mL), both cells were harvested in lysis buffer (Pierce, Rockford, IL) and equal amounts of protein were subjected to 12% SDS-PAGE. After gel electrophoresis, the proteins were transferred to polyvinylidene difluoride membranes (Immobilon PVDF; Millipore). The membranes were blocked for 1 h at room temperature in 5% non-fat dry milk in tris buffered saline (TBS) containing 0.05% Tween 20, followed by an overnight incubation at 4°C with primary antibodies. The membranes were then incubated with horseradish peroxidase-labeled anti-rabbit secondary antibody (Chemicon) for 1hr at room temperature. Peroxidase activity was detected via the chemiluminescence (SuperSignal West Femto luminol substrate and peroxide buffer; Pierce). Primary antibodies, including anti-JAK2, anti-p-JAK2, anti-STAT3, anti-p-STAT3, anti-MEK, anti-p-MEK, anti-ERK, anti-p-ERK, anti-NF-κB, anti-iNOS, anti-cyclin-D1, anti-Bcl-2 and anti-GAPDH (Cell Signaling technology), were used.

### Reverse transcription PCR (RT-PCR) analysis

The mRNA levels of IL-6 in HCCLM3 cells were detected as follows. Total RNA was extracted following the manufacturer's protocol (Invitrogen). Real-time RT-PCR analysis for quantification was performed using an SYBR Premix Ex Taq™ (perfect real time; TaKaRa). The relative mRNA expression was normalized to that of β-actin. The relative amount of tissue mRNA was standardized by the amount of β-actin mRNA, and expressed as−ΔCT=CT (factor)−CT (β-actin). The ratio of the number of mRNA copies to the number of β-actin mRNA copies was then calculated as 2–ΔCT×K, where K is a constant. The primers for IL-6 was used as follows: 5′-GAACTCCTTCTCCACAAGCG-3′ (forward) and 5′-TTTTCTGCCAGTGCCTCTTT-3′ (reverse).

### Nitric oxide (NO) measurement

Direct NO measurement was performed at 37 °C using the Apollo 4000 (WPI Inc., USA), which is an optically isolated multi-channel free radical analyzer with an NO selective membrane. After 24 and 48 hr culturing of HCCLM3-wt or HCCLM3-IL6(-) with Sorafenib (10 umol/L) and/or IFNα (50000 U/mL), the cell monolayer was washed with PBS and immersed in 1 mL of PBS. The real-time acquisition of NO production through a single-board computer that displays the experimental data.

### Animals, orthotopic tumor model, and treatment *in vivo*

Male BALB/c nu/nu nude mice, weighing approximately 20 g (Shanghai Institute of Materia Medica, Chinese Academy of Sciences, Shanghai, PR China), were housed in laminar flow cabinet under specific pathogen-free condition and used at the age of 6 wk. The mice were cared for and handled in accordance with the National Institutes of Health Guidelines for the Care and Use of Laboratory Animals. The experimental protocol was approved by the Shanghai Medical Experimental Animal Care Committee.

The HCC tumor model was established by the orthotropic implantation of a histologically intact tumor tissue derived from HCCLM3-wt and HCCLM3-IL-6(-) cell lines. When the average tumor volume had reached 100 mm^3^ (first week of tumor implantation), IFN-α (1.5 × 10^7^ U/kg), sorafenib (30 mg/kg), IFN-α+Sorafenib, and normal saline (NS) were injected subcutaneously every day for six wk to observe their effects on tumor growth. The tumor size was assessed using the formula width × length × depth × π/6.

## Abbreviations

HCC: Hepatocellular carcinoma
TALEN: transcription activator-like effector nucleases
BMOL: bipotential murine oval liver
CCK-8: the cell counting kit-8
NO: nitric Oxide
NS: normal saline
